# Draft Genome Sequence of *Leifsonia* sp. Strain NCR5, a Rhizobacterium Isolated from Cadmium-Contaminated Soil

**DOI:** 10.1128/genomeA.00520-17

**Published:** 2017-06-08

**Authors:** Eleonora Egidi, Jennifer L. Wood, Edward M. Fox, Wuxing Liu, Ashley E. Franks

**Affiliations:** a Department of Physiology, Anatomy and Microbiology, La Trobe University, Bundoora, Victoria, Australia; b CSIRO Agriculture and Food, Werribee, Victoria, Australia; c Key Laboratory of Soil Environment and Pollution Remediation, Institute of Soil Science, Chinese Academy of Sciences, Nanjing, China

## Abstract

We report here the draft genome sequence of *Leifsonia* sp. strain NCR5, a Gram-positive actinomycete isolated from *Carpobrotus rossii* (Haw.) Schwantes rhizosphere. The *de novo* genome of *Leifsonia* sp. strain NCR5 was assembled with 69 scaffolds and a G+C content of 69%, was 4.2 Mb in length, and contained 3,952 coding sequences.

## GENOME ANNOUNCEMENT

The genus *Leifsonia* encompasses aquatic (1), endophytic (2), and plant-pathogenic (3) species of Gram-positive bacteria. Members from this genus are considered rare among the actinomycetes (4). Due to the difficulty in cultivating these fastidious organisms, only a few *Leifsonia* sp. representative genomes are currently available (5). We report here the draft genome sequence of *Leifsonia* sp. strain NCR5, a rhizosphere inhabitant obtained from the heavy-metal hyperaccumulator *Carpobrotus rossii* (Haw.) Schwantes grown in a cadmium-contaminated environment. Information on the genetic potential of this important member of the soil biosphere will provide further insights into the physiological and metabolic abilities of rhizosphere-associated actinomycetes, as well as open possible future applications in microbe-mediated bioremediation of heavy-metal-contaminated soils.

The strain was grown in cell culture, and the total genomic DNA was extracted from purified NCR5 colonies and converted to sequencing libraries using the Nextera XT kits (Illumina). Libraries were normalized and pooled before sequencing on an Illumina MiSeq with 2 × 300 paired-end reads. The A5-miseq pipeline (6) was used to perform read trimming and correction, contig assembly, crude scaffolding, misassembly correction, and final scaffolding. No putative misassemblies were detected with this method. The resulting genome size of *Leifsonia* sp. strain NCR5 is 4,222,396 bp, and the mean G+C content is 69% (101-fold median coverage), with a final number of contigs of 69 and *N*_50_ value of 150,950 bp.

Gene annotation was performed using RAST version 2.0 (7), resulting in 3,952 coding sequences and 51 RNA-encoding genes; the closest genome to NCR5 was that of *Leifsonia xyli* subsp. *xyli* strain CTCB07. The *Leifsonia* sp. NCR5 genome harbors several genes associated with resistance to a wide range of heavy metals. In particular, we retrieved 10 genes coding for various arsenic resistance proteins, such as arsenate reductases and the arsenical resistance protein ACR3, 1 gene coding for a mercuric reductase, and 2 genes related to cobalt-zinc-cadmium resistance (*czcD* and *merR*). Furthermore, the NCR5 genome is equipped with genes associated with bacterial motility and chemotaxis, as well as organic acids, aromatic compounds, and carbohydrate metabolism, suggesting the potential for this strain to colonize the plant root and utilize numerous root exudates. Possible plant growth-promoting abilities are implied by the occurrence in the genome of genes related to siderophore and trehalose biosynthesis.

### Accession number(s).

This whole-genome shotgun project has been deposited at DDBJ/ENA/GenBank under the accession no. NCSR00000000.
